# The Clinical Utility of Systemic Immune-Inflammation Index Supporting Charlson Comorbidity Index and CAPRA-S Score in Determining Survival after Radical Prostatectomy—A Single Centre Study

**DOI:** 10.3390/cancers14174135

**Published:** 2022-08-26

**Authors:** Piotr Zapała, Karolina Garbas, Zbigniew Lewandowski, Łukasz Zapała, Aleksander Ślusarczyk, Cezary Ślusarczyk, Łukasz Mielczarek, Piotr Radziszewski

**Affiliations:** 1 Clinic of General, Oncological and Functional Urology, Medical University of Warsaw, Lindleya 4, 02-005 Warsaw, Poland; 2 Department of Epidemiology, Medical University of Warsaw, Oczki 3, 02-007 Warsaw, Poland; 3 Second Department of Urology, Centre of Postgraduate Medical Education, Cegłowska 80, 01-809 Warsaw, Poland

**Keywords:** prostate cancer, life expectancy, systemic immune-inflammation index, Charlson comorbidity index, CAPRA-S, early survival

## Abstract

**Simple Summary:**

Radical treatment of prostate cancer (PCa) provides excellent oncological outcomes. However, curative treatment of primary PCa, as well as salvage treatment of biochemical recurrence after radical treatment, requires at least a 10-year life expectancy to be beneficial. To provide an accurate selection for active treatment, several tools evaluating individual life expectancy have been developed. Our retrospective study aimed to determine the utility of the systemic immune-inflammation index (SII) in predicting early survival when used as an adjunct to CAPRA-S and Charlson comorbidity index (CCI) scores in non-metastatic PCa. We confirmed the SII as an independent predictor of survival. We have also validated the SII as a supplement to scoring systems when stratifying the risk of early mortality. In the setting of patients that might require salvage treatment, supplementing comorbidity status with the SII provided accurate discrimination of survival. The SII seems then to be useful when estimating life expectancy in patients with non-metastatic PCa.

**Abstract:**

The selection of candidates for the curative treatment of PCa requires a careful assessment of life expectancy. Recently, blood-count inflammatory markers have been introduced as prognosticators of oncological and non-oncological outcomes in different settings. This retrospective, monocentric study included 421 patients treated with radical prostatectomy (RP) for nonmetastatic PCa and aimed at determining the utility of a preoperative SII (neutrophil count × platelet count/lymphocyte count) in predicting survival after RP. Patients with high SIIs (≥900) presented significantly shorter survival (*p* = 0.02) and high SIIs constituted an independent predictor of overall survival [HR 2.54 (95%CI 1.24–5.21); *p* = 0.01] when adjusted for high (≥6) age-adjusted CCI (ACCI) [HR 2.75 (95%CI 1.27–5.95); *p* = 0.01] and high (≥6) CAPRA-S [HR 2.65 (95%CI 1.32–5.31); *p* = 0.006]. Patients with high scores (ACCI and/or CAPRA-S) and high SIIs were at the highest risk of death (*p* < 0.0001) with approximately a one-year survival loss during the first seven years after surgery. In subgroup of high CAPRA-S (≥6), patients with high ACCIs and high SIIs were at the highest risk of death (*p* <0.0001). Our study introduces the SII as a straightforward marker of mortality after RP that can be helpful in pre- and postoperative decision-making.

## 1. Introduction

Prostate cancer (PCa) is the second most prevalent cancer in males, with 1.4 million patients diagnosed in 2020 worldwide [1] and the lifetime risk of PCa-specific death reaching 2.5–4% [2]. Due to favourable survival in patients with untreated localized disease, active treatment is considered beneficial only in patients with a life expectancy of at least 10 years [3]. The majority of comorbid, elderly patients will die from competing causes and the impact of their tumours’ characteristics on their 10-year overall survival is unclear [4], which necessitates the prediction of life expectancy in patients with PCa as a part of clinical practice [5,6,7,8]. Simultaneously, stratifying survival in patients treated with curative intent remains challenging due to favourable prognoses and extensive censoring in observational studies, leaving the definition of the patient at risk of early death yet to be specified. The European Association of Urology mentions the commonly used Charlson comorbidity index (CCI) and the age-adjusted Charlson comorbidity index (ACCI) as convenient measures of non-cancer-specific death risk [8,9], whereas the Cancer of the Prostate Risk Assessment Postsurgical score (CAPRA-S) based on pathological characteristics has been extensively validated to predict biochemical recurrence [10] and cancer-specific mortality after radical prostatectomy (RP) [11].

In recent years, complete blood count inflammatory markers have been sequentially introduced as predictors of survival in localized renal cell cancer [12,13,14] and bladder cancer [15,16], as well as in radio-recurrent [17] and castration-resistant prostate cancer [18]. The systemic immune-inflammation index (SII) is a novel, recently developed blood count-derived marker that has shown particularly promising predictive properties in the cohort with non-metastatic prostate cancer [19], which constitutes a population with excellent early survival. In otherwise treatment-naïve, nonmetastatic patients, a high SII was associated with a risk of biochemical recurrence (BCR) after radical prostatectomy independently from postoperative staging and grading [20]. In a similar analysis of a large cohort by Rajwa et al., a high SII was an independent predictor of BCR both in preoperative and postoperative settings [19]. In the retrospective cohort of 214 patients with radio-recurrent PCa, a high SII was an independent prognostic factor for overall and cancer-specific survival both in the preoperative and postoperative adjustment settings [17]. Although data on radio-recurrent patients suggest an impact of SII on survival, the evidence of long-term outcomes in patients treated a priori with RP is limited to the association with biochemical recurrence.

The study aimed to determine the utility of the systemic immune-inflammation index in predicting early survival when used as an adjunct to CAPRA-S and CCI scores in patients with non-metastatic prostate cancer.

## 2. Materials and Methods

### 2.1. Patients

Patients treated with radical prostatectomy in the years 2012–2018 for clinically nonmetastatic prostate cancer in a single tertiary centre were included in the analysis. All patients underwent routine metastatic screening as recommended by a relevant edition of the European Association of Urology Guidelines. This consisted of at least a bone scan in intermediate-risk disease and a bone scan combined with cross-sectional abdominopelvic imaging in high-risk disease. Patients treated with salvage intention or neoadjuvant hormonotherapy were excluded.

### 2.2. Data Collection

Clinical and pathological data were retrieved from the department database. Blood cell counts were obtained from routinely performed preoperative evaluations during the hospitalization. The systemic immune–inflammation index (SII) was calculated by multiplying the neutrophil count by the platelet count and dividing the score by the lymphocyte count as previously [17,19,20]. The Charlson comorbidity index was calculated based on its last update [21] and included 19 conditions with scores assigned based on their severity. For age-adjusted CCI, beginning at the age of 50 years, one point was added to the CCI for each subsequent decade. The cut-off for the high score was set at 4 or greater for CCI, or 6 or greater for age-adjusted CCI. Both calculations included prostate cancer (2 points) as a covariable. The cut-off selection for CCI and age-adjusted CCI (high vs. low) was based on cut-offs used in previous localized PCa cohorts, with a correction for PCa which was included in our calculations [22,23]. The CAPRA-S score included prostate-specific antigen (PSA), pathologic Gleason score, positive surgical margins, extracapsular extension, seminal vesicle invasion, and lymph node invasion. CAPRA-S groups were defined as previously with high risk defined as 6 or greater [10].

Survival follow-up data were retrieved from the Central Statistical Office. The study was approved by the Ethics Committee of the Medical University of Warsaw (nr AKBE/58/2022; 21 February 2022).

### 2.3. Statistical Analysis

All analyses were performed using SAS 9.4 software (SAS Institute, Cary, NC, USA). Continuous and qualitative variables were compared utilizing Mann–Whitney’s U-test and Fisher’s exact test, respectively. The cut-off for SII was set at a value maximizing the difference between overall survival probabilities, rounded to the nearest 100 units. Overall survival estimates were calculated using the Kaplan–Meier method. The log-rank test was used for comparisons of survival curves between predefined risk groups. For multiple comparisons, a log-rank test with an adjustment for multiple comparisons was implemented. Cox regression models were utilized for multivariate analysis issues to determine independent predictors of early death after RP. For clinical convenience, a quantitative evaluation of survival was performed utilizing restricted mean survival time. The threshold for significance was set at *p* < 0.05.

## 3. Results

### 3.1. Baseline Characteristics

A total of 421 patients were included in the analysis. All patients were white, non-Hispanic men. High SIIs (≥900) were present in 218 patients (51.80%). In general, a high SII was not associated with significantly higher pre- and postprostatectomy grading or adverse pathological features (APF). The baseline characteristics of the patients stratified by SII (high vs. low) are summarized in Table 1.

### 3.2. Survival Predictors

The median follow-up was 69 months (95%CI 66.5–71.9 months). In the analysed period, a total of 33 deaths (7.86%) were reported.

To determine the optimal SII cut-off, several thresholds were tested (Table 2). The value of 900 provided the maximum difference between survival maintaining statistical significance and was used for stratification purposes.

**Figure 1:**
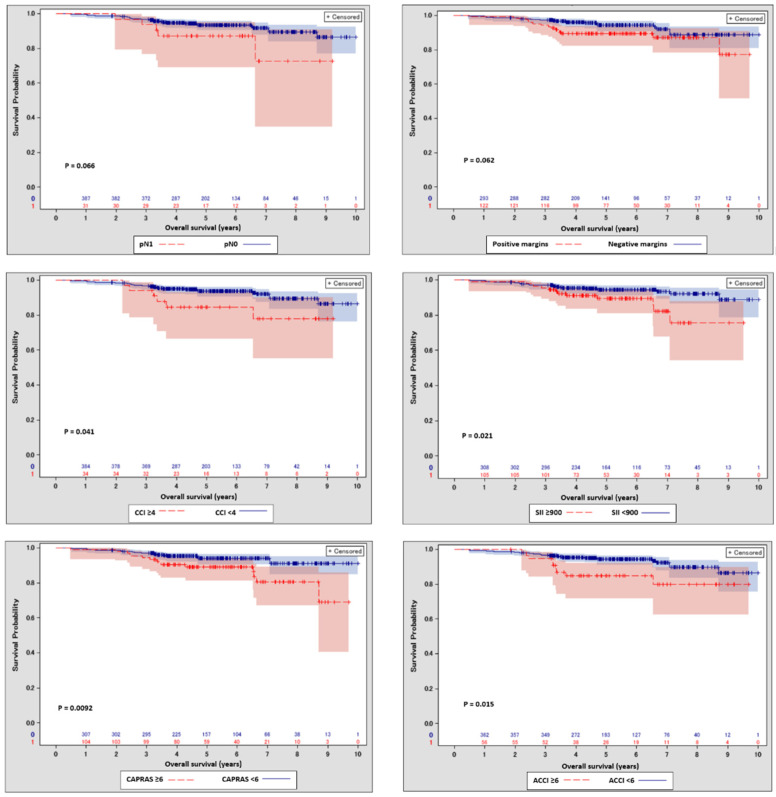
Kaplan-Meier curves with 95% confidence intervals for patients stratified with margin and nodal status, age-unadjusted Charlson comorbidity index (≥4) and age-adjusted Charlson comorbidity index (≥6), systemic immune-inflammation index (≥900) and CAPRA-S (≥6).

In a univariate survival analysis using baseline clinical and pathological factors, high (≥4) CCI (*p* = 0.041), high (≥6) ACCI (*p* = 0.015), high (≥6) CAPRA-S score (*p* = 0.0092) and high (≥900) SII (*p* = 0.021) were associated with a higher risk of death. Positive surgical margin status (*p* = 0.062) and lymph node involvement (*p* = 0.066) also showed a tendency for statistical significance (Figure 1), whereas pre-and postoperative grading as well as local pathological staging, preoperative PSA, and CAPRA score revealed no significant association with survival (data not shown). CAPRA-S, CCI and ACCI revealed modest concordance in the univariate prediction of death (c-index 0.59, 0.55 and 0.58, respectively).

**Figure 2:**
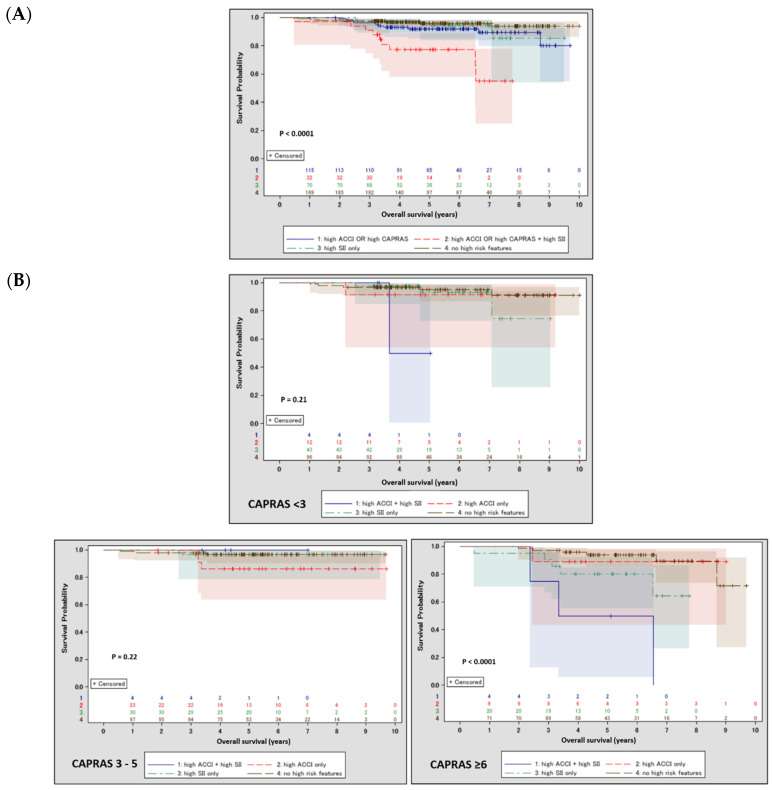
Kaplan-Meier curves with 95% confidence intervals depicting survival after radical prostatectomy depending on ACCI, CAPRA-S and SII. (**A**) Survival in patients stratified by the combination of either score with SII—entire cohort; (**B**) survival in patients stratified by the combination of ACCI with SII depending on the CAPRA-S risk group.

To provide a rationale for combining scoring systems with SII, two multivariate models were built (Table 3) confirming high CAPRA-S score, high ACCI or CCI and high SII as independent predictors of overall mortality. Stratification combining both scoring systems with an SII yielded significantly worse survival in patients presenting at least one high score simultaneously with a high SII (Figure 2A). CAPRA-S risk groups were then used for a subgroup analysis (Figure 2B), which revealed that patients with high SIIs coexisting with high ACCIs were at the highest risk of mortality in high CAPRA-S (≥6).

**Figure 3:**
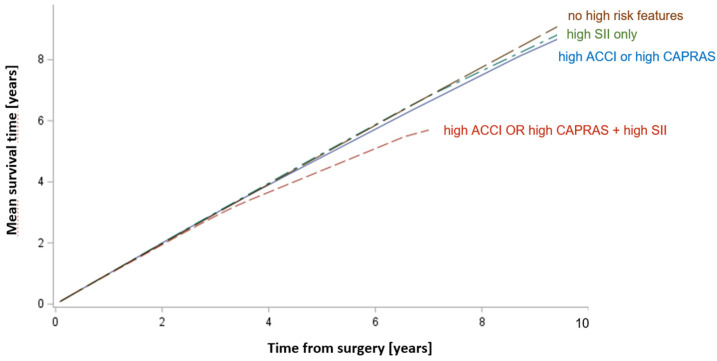
The mean survival time corresponding to the increasing length of follow-up among the four groups of patients.

A quantitative analysis of survival yielded a clinically significant difference in survival between predefined subgroups, suggesting approximately a one-year survival loss in patients combining high SIIs with a single high score (CAPRA-S or CCI) achieved after 7 years when compared with other groups (Figure 3). In an explanatory analysis utilizing multiple comparisons, we found no significant differences in survival between patients presenting high SII only, a high score (ACCI or CAPRAS) and no risk features (comparing each of the groups with one another). We found however a significant difference in survival between patients presenting a high score (ACCI or CAPRAS) combined with high SII and patients from each of the three remaining groups—with high SII only (*p* = 0.012), with a high score (ACCI or CAPRAS) only (0.046) and with no risk features (*p* = 0.013).

## 4. Discussion

In this study, we present the systemic immune-inflammation index as a potential adjunct marker in predicting early death in patients treated with RP due to non-metastatic prostate cancer. We have validated the SII as an independent predictor of survival and developed a proposal for consecutive supplementation of the CAPRA-S score and Charlson comorbidity index with the SII.

The question of life expectancy in patients considered for radical treatment comes from existing evidence that men with less than 15 years of predicted survival are unlikely to benefit [24,25]. The clinical implementation of magnetic resonance imaging and novel markers, including next-generation PSA-based tests, improved the detection of clinically significant cancer and reduced overdiagnosis [26]. In turn, overtreatment reduction requires a deep insight into expected survival in a “risk and benefits” manner. Estimating individual life expectancy remains however a complex task which requires taking into account not only age [5] and co-morbidity [5,8,21], but also the risk profile of the cancer in itself [10,11], which, when neglected, can lead to undertreatment.

Increasing the utility of salvage treatment in recurrent PCa broadens the challenge of estimating life expectancy for patients who have already completed radical treatment. It has been shown that 20% of patients undergoing salvage RP die of other causes [27], whereas patients after RP with a favourable PSA doubling time (PSA-DT) and low postprostatectomy grading who experience biochemical recurrence might not benefit from salvage radiotherapy in a follow-up of 10 years [28]. Finally, even patients with postoperative PSA-DT shorter than 10 months who choose deferred radiotherapy can achieve an overall survival (OS) of more than 200 months [29]. Due to the following reasons, the paradigm of 10-year expected survival has been re-introduced, this time in patients considered for salvage treatment [30].

CAPRA-S has been developed as a straightforward calculator of biochemical recurrence after RP [10]. Extensive validation of the tool has yielded satisfactory accuracy and CAPRA-S has managed to outperform the Stephenson nomogram decision curve analysis when predicting BCR [11]. Interestingly, the good performance of CAPRA-S has also translated into the prediction of cancer-specific survival with a high concordance of 0.85 [11]. In our study, we have confirmed CAPRA-S as an independent predictor of OS. In the first place, however, we used CAPRA-S as primary stratification before adjusting for comorbidities and haematological markers to simulate the salvage treatment setting when validating the CCI, ACCI and SII.

The Charlson comorbidity index has been developed [9,21] and validated as a stratification of comorbidities influencing overall survival in multiple clinical settings [7,8,22,31]. Although the CCI has no value in prognosing PCa-specific mortality [31], it has proved to be a powerful predictor of overall mortality in nonmetastatic PCa, regardless of the D’Amico risk group [31]. In our study, patients with ACCIs ≥ 6 presented significantly worse OS and a high CCI/ACCI constituted an independent predictor of death in the multivariate analysis. Patients at postprostatectomy risk of receiving salvage therapy (CAPRA-S score higher than 5) were significantly less likely to survive if presenting a high ACCI at baseline. Since high comorbidity can translate into less apparent oncological benefits, we believe that the ACCI in the post-RP setting might tip the balance towards deferred salvage treatment or watchful waiting.

The systemic immune-inflammation index is a novel blood count-derived marker that has been introduced as a prognosticator in metastatic castration-resistant patients [32,33,34]. In the study of Lolli et al., an SII ≥ 535 predicted OS independently from the ECOG status, previous enzalutamide treatment and the presence of visceral metastases with HR = 1.8 [32]. The study of Fan et al. yielded similar outcomes, although with a lower threshold of SII ≥ 200 (overall mortality HR = 4.7; *p* = 0.001) [33].

Although in a preprostatectomy setting, the SII has not been confirmed as a predictor of unfavourable biopsy outcomes [35], recently the SII has been extensively validated in patients who have already undergone radical treatment. Similarly to other haematological markers, the SII can be easily retrieved and calculated from a full blood count, which is an obligatory part of preoperative laboratory evaluations. In a large multicenter cohort of patients treated with RP [19], high preoperative SIIs (≥620) were independently associated with extracapsular extension (odds ratio [OR] 1.16, *p* = 0.041), non-organ confined disease (OR 1.18, *p* = 0.022), adverse pathology (OR 1.36, *p* < 0.001) and upgrading at RP (OR 1.23, *p* < 0.001). A high SII also constituted an independent predictor of BCR in the preoperative model (HR 1.34, *p* < 0.001), but not in the postoperative model (*p* = 0.078). Another multicenter study by Rajwa et al. [17] included 214 patients with radio-recurrent PCa treated with salvage prostatectomy. Both preoperative and postoperative models confirmed a high SII (≥730) as an independent prognostic factor for cancer-specific survival (HR = 10.7, *p* = 0.039 and HR = 22.11, *p* = 0.036, respectively) and overall survival (HR = 8.57, *p* < 0.001 and 5.98, *p* = 0.006, respectively). Implementing the SII as an adjunct to previous preoperative models might therefore aid decision-making on salvage prostatectomy, especially in comorbid patients who are concerned about SRP harms.

In our study, we failed to validate a high SII as a preoperative predictor of adverse pathology. The discrepancy with previous studies might come from different characteristics of PCa in our cohort. The majority of the patients from the benchmark study by Rajwa et al. presented low pre- (61% grade group [GG] 1, 23% GG2) and postprostatectomy grading (32% GG1, 36% GG2). Nodal involvement (<2%), as well as an extracapsular extension (23%) and seminal vesicles invasion (6%), were uncommon in the development set [19]. In contrast, our patients were less likely to present with GG1 or GG2 in biopsy (38.73% and 31.37%, respectively) and post-RP specimen (13.94% and 39.66%, respectively), whereas the prevalence of pT3a and pT3b was considerably higher (42.37% and 14.52%, respectively). Noteworthy, previous cohorts with comparable prevalences of adverse pathology, such as the one analysed by Bravi et al., also yielded negative outcomes, failing to validate the association of NLR with adverse pathology [36]. This might suggest that the prognostic performance of particular haematological markers when predicting APF might depend on the risk profile of the cohort.

In our study, we managed to confirm a high SII as an independent predictor of overall survival when adjusted for adverse pathology using the CAPRA-S score and comorbidities using the CCI. Although isolated use of CAPRA-S and the CCI showed only limited value in predicting OS in short follow-ups, patients presenting with a high outcome of at least one of the scorings simultaneously with a high SII were particularly predisposed to early mortality. Finally, a high CCI supplemented with SII ≥ 900 provided genuine discrimination of overall mortality in patients from the high (>5) CAPRA-S risk group. The proposed stratifications might be easily utilised especially in a post-RP setting when deciding on salvage treatment.

The study has several limitations. Firstly, this is an observational analysis with retrospective data collection and limited follow-up. Although a detailed evaluation of comorbidities constitutes a routine workup of a patient before surgery, the CCI was evaluated based on reported comorbidities without a uniform survey procedure. Adjuvant or salvage treatment of the patients after RP was at a particular clinician’s discretion and discrepancies in oncological follow-up might have theoretically confounded the survival outcome. Data regarding post-RP radiotherapy could not be collected, limiting bias detection and evaluation. Since this was a single-centre study, selection bias cannot be ruled out and some results might not be transferable to every clinical community. On the other hand, patient-tailored treatment and follow-up according to best local practice do not affect the major outcomes of this observational analysis. The primary aim of the study was to evaluate overall survival, which is described mainly by other-cause mortality in the post-RP setting; however, the lack of cancer-specific follow-up data prevented us from separating the contribution of cancer-dependent mortality.

## 5. Conclusions

Our study introduces the high systemic immune-inflammation index as a potential marker of survival in patients treated with radical prostatectomy for nonmetastatic prostate cancer. We have validated the SII as an adjunct to the commonly used CAPRA-S score and the Charlson comorbidity index. We estimated that patients combining high SIIs with high age-adjusted CCIs and/or high CAPRA-S survive approximately one year less during the first seven years after surgery. We have also confirmed the SII as useful when stratifying the risk of death in patients that might be potential candidates for salvage treatment (intermediate–high and high CAPRA-S). We believe that the implementation of the SII into already used prognostic tools might be of clinical value, especially when improving the selection of candidates for the radical treatment of prostate cancer.

## Figures and Tables

**Table 1:** Baseline pre- and postprostatectomy characteristics of patients stratified by SII (high vs. low).

Variable		Overall	SII < 900	SII ≥ 900	*p*
Clinical baseline characteristics					
PSA (ng/mL, median, IQR)		7.40 (6.7)	7.5 (6.7)	7 (5.5)	0.44
cT (*n*%)	cT1	149 149 (37.44%)	101 (34.95%)	45 (43.27%)	0.17
	cT2	244 (61.31%)	183 (63.32%)	59 (56.73%)	
	≥cT3	5 (1.26%)	5 (1.73%)	0	
Biopsy grade group (*n*,%)	1	158 (38.73%)	115 (38.33%)	40 (38.83%)	0.73
	2	128 (31.37%)	90 (30%)	37 (35.92%)	
	3	54 (13.24%)	41 (13.67%)	13 (12.62%)	
	4	47 (11.52%)	37 (12.33%)	9 (8.74%)	
	5	21 (5.15%)	17 (5.67%)	4 (3.88%)	
CCI (*n*,%)	2	308 (73.33%)	224 72.49%)	79 (74.53%)	0.37
	3	78 (18.57%)	56 (18.12%)	22 (20.75%)	
	4	27 (6.43%)	24 (7.77%)	3 (2.83%)	
	5	4 (0.95%)	3 (0.97%)	1 (0.94%)	
	6	3 (0.71%)	2 (0.65%)	1 (0.94%)	
Age (years, median, IQR)		65 (8)	65 (8)	64 (8)	0.57
Postprostatectomy specimen					
Prostatectomy grade group (*n*,%)	1	58 (13.94%)	46 (14.98%)	10 (9.62%)	0.30
	2	165 (39.66%)	116 (37.79%)	47 (45.19%)	
	3	89 (21.39%)	62 (20.20%)	26 (25%)	
	4	62 (14.90%)	50 (16.29%)	12 (11.54%)	
	5	42 (10.10%)	33 (10.75%)	9 (8.65%)	
pT (*n*,%)	pT2	241 (57.93%)	171 (55.70%)	65 (62.50%)	0.41
	pT3	173 (41.59%)	134 (43.65%)	39 (37.50%)	
	pT4	2 (0.48%)	2 (0.65%)	0	
pN (*n*,%)	pN0	181 (43.20%)	134 (43.51%)	45 42.45%)	0.20
	pN1+	31 (7.40%)	27 (8.77%)	4 (3.77%)	
	pNx	207 (49.40%)	147 (47.73%)	57 (53.77%)	
EPE (*n*,%)		175 (42.37%)	136 (44.59%)	39 (37.86%)	0.25
SVI (*n*,%)		61 (14.52%)	48 (15.53%)	13 (12.26%)	0.52
PSM (*n*,%)		123 (29.50%)	91 (29.55%)	32 (30.77%)	0.81

SII—systemic immune-inflammation index; PSA—prostate-specific antigen [ng/mL]; cT—clinical staging; CCI—Charlson comorbidity index; pT—pathological local staging; pN—pathological nodal staging; EPE—extracapsular extension; SVI—seminal vesicles involvement; PSM—positive surgical margins.

**Table 2:** Differences in survival probabilities derived using different SII cut-offs.

SII Cut-Off	Cut-Off Percentile	8-Year Survival Probability Difference	Log-Rank *p*-Value
600	50	7.28%	0.1525
700	60	9.93%	0.1259
800	68	12.75%	0.0789
900	75	16.58%	**0.0206**
1000	80	16.72%	0.056

SII—systemic immune-inflammation index. *p*-value in the bold is the only significant.

**Table 3:** The association of SII with overall survival in patients treated with radical prostatectomy for nonmetastatic prostate cancer-multivariable Cox regression analyses, including categorized SII, CAPRA-S and age-adjusted Charlson comorbidity index (ACCI) or age-unadjusted Charlson comorbidity index (CCI).

Variable	HR (95% CI)	*p*
multivariate model 1 (c index = 0.67)		
CAPRA-S ≥ 6	2.67 (1.33–5.35)	0.006
CCI ≥ 4	2.79 (1.14–6.84)	0.025
SII ≥ 900	2.59 (1.26–5.31)	0.009
multivariate model 2 (c index = 0.67)		
CAPRA-S ≥ 6	2.65 (1.32–5.31)	0.006
ACCI ≥ 6	2.75 (1.27–5.95)	0.01
SII ≥ 900	2.54 (1.24–5.21)	0.01

SII—systemic immune inflammation index; HR—hazard ratio; CI–confidence interval; CAPRA—The Cancer of the Prostate Risk Assessment score; CCI—Charlson comorbidity index; ACCI—age-adjusted Charlson comorbidity index.

## Data Availability

The data presented in this study are available on request from the corresponding authors.
